# Osteonecrosis of Femoral Head, An Overlooked Long-Term Complication after Paraquat Intoxication: A Retrospective Cohort Study

**DOI:** 10.1038/s41598-020-65756-w

**Published:** 2020-06-01

**Authors:** Ming-Jen Chan, Chien-Chang Huang, Ching-Chih Hu, Wen-Hung Huang, Ching-Wei Hsu, Tzung-Hai Yen, Cheng-Hao Weng

**Affiliations:** 1 Kidney Research Center, Department of Nephrology, Linkou Chang Gung Memorial Hospital, Taoyuan, Taiwan; 2 Clinical Poison Center, Chang Gung Memorial Hospital, Linkou Medical Center, Taoyuan, Taiwan; 3 Chang Gung University College of Medicine, Taoyuan, Taiwan; 4 Graduate Institute of Clinical Medical Sciences, College of Medicine, Chang Gung University, Taoyuan, Taiwan; 5 Department of Hepatogastroenterology and Liver Research Unit, Chang Gung Memorial Hospital, Keelung, Taiwan

**Keywords:** Diseases, Health care, Medical research, Nephrology

## Abstract

With increasing numbers of patients surviving acute intoxication phase, long-term complication after paraquat intoxication is a topic worth exploring, such as osteonecrosis (ON) of femoral head. We reviewed 86 paraquat-intoxicated survivors between 2000 and 2012 in Chang Gung Memorial Hospital, a 3700-bed tertiary hospital in Taiwan. With all the patients underwent same detoxification protocol in the acute stage, 17.4% of paraquat poisoning survivors developed ON of femoral head requiring surgery during follow up. Most of ON episodes occurred within 2 to 4 years after paraquat intoxication and then plateau after 6 years. ON patients exhibited higher SOFA scores than non-ON patients (2.80 ± 2.14 vs. 1.76 ± 1.52, p = 0.028). Furthermore, AKIN scores are also higher in the ON patients than non-ON patients (0.87 ± 1.13 vs. 0.38 ± 0.74, p = 0.040). Multivariate logistic regression showed higher AKIN score and higher partial pressure of carbon dioxide in the blood 48 hours after admission significantly predicted ON of femoral head after paraquat intoxication (p = 0.002 and p = 0.006 respectively). Larger studies with longer follow-up durations are warranted to confirm our finding.

## Introduction

Costing less than 5 U.S dollars per liter, paraquat is a common contact herbicide with extremely high toxicity in Taiwan. Deliberately or unintentionally ingestion of paraquat is common^1^. Paraquat consumption is fatal in 60–80% of cases due to extreme toxicity. 40 mL of a 24% paraquat solution is enough to cause multiple organ failure and mortality within days^2^. Paraquat is absorbed quickly after ingestion and is mostly excreted in the urine without further metabolism within 12–24 hours. Paraquat intoxication leads to acute lung injury, multiple organ failure, and acute kidney injury^3^. We used a standard detoxification protocol including charcoal hemoperfusion, pulse therapies with methylprednisolone and cyclophosphamide, and extended treatment with dexamethasone to treat all paraquat intoxicated patients^4,5^. This protocol has been reviewed and recommended by the Cochrane Injuries Group as beneficial in cases of lung fibrosis caused by paraquat^6^. Most of previous literature reported acute poisoning epidemiology, clinical symptoms, acute complication and treatment of paraquat. Literature focusing on long-term follow-up after the paraquat poisoning is still very scarce. With increasing numbers of patients surviving acute intoxication phase, long-term complication after paraquat intoxication is a topic worth exploring. We noticed several patients developed osteonecrosis (ON) of femoral head during paraquat intoxication long-term follow up. Our retrospective study was inspired by this observation.

ON of femoral head, or avascular necrosis of femoral head, is a heavy burden for its victim due to its debilitating nature, physical and phycological alike^7^. It is a progressive pathological condition caused by insufficient blood supply to the subchondral bone area with subsequent osteocyte death. Though exact mechanism is still under investigation, bone vasculature compromise causing marrow infarction with subsequent structure collapse is common to most proposed etiologies. Besides, both direct damage to osteocytes (e.g., by toxin production) and indirect damage (e.g., due to disorders of fat metabolism or hypoxia) may lead to ON^8–11^. A variety of factors contribute to ON of femoral head, including traumatic and nontraumatic^9,12^. Glucocorticoid administration and alcohol use account for more than 80% of nontraumatic ON of femoral head^13^. Severe lung injury and hypoxia due to paraquat intoxication are frequently observed^2,14^. Paraquat intoxication would also induce oxidative stress, which is currently researched as one of the factors of ON^10,11,15–17^. As most of the long-term complications of paraquat have been ignored, research about ON of femoral head after paraquat intoxication is also very rare. There are only two previous studies reported ON of femoral head after paraquat intoxication, but both are case reports^18,19^. There is no retrospective study for paraquat intoxication related ON of femoral head till this date. In this study, we investigated the predictors of ON of femoral head after paraquat intoxication.

## Results

### Subject characteristics

As shown in Table 1, the patient is 35.22 ± 12.42 years old, with 65 (75.5%) men and 21 (24.5%) women. Average of estimated paraquat ingestion amount is 56.27 mL. Fifteen patients experienced ON of femoral head (17.4%). Major depression disorder and alcoholism were prevalent in both ON and non-ON group. Median duration of steroid treatment was 28 days. Cumulative steroid dose (prednisone equivalent for all oral and intravenous administration) is 6.47 ± 5.27 g. Table 2 demonstrated basic data of ON patients. All of the ON patients had advanced Association of Research Circulation Osseous (ARCO) stage^20^. Bilateral ON of femoral heads are noted in 5 patients. All ON episodes occur in femoral head. Pathology report is available in 10 patients and all compatible with ON. ON patients exhibited higher SOFA _48-h_ scores than non-ON patients (2.80 ± 2.14 vs. 1.76 ± 1.52, p = 0.028). Furthermore, AKIN _48-h_ scores are also higher in the ON patients than non-ON patients (0.87 ± 1.13 vs. 0.38 ± 0.74, p = 0.040). The follow up duration is also shorter in the ON group than non-ON group (2.91 ± 2.43 vs. 10.80 ± 5.07, p < 0.001). Though not reaching statistically significance, ON patient has higher first day urine paraquat level (35.85 ± 20.08 vs. 27.16 ± 21.21, p = 0.150), higher first day creatinine level (1.81 ± 0.81 vs. 1.29 ± 0.87, p = 0.167), higher PaCO_2 48-h_ (44.28 ± 24.80 vs. 37.50 ± 7.99, p = 0.059), lower PaO_2 48-h_ patients (66.33 ± 15.92 vs. 73.30 ± 18.84 p = 0.186), and higher HCO_3_^−^_first day_ (23.88 ± 3.99 vs. 22.01 ± 3.37, p = 0.062) than their non-ON counterparts.

**Table 1:** Patients’ demographic data and clinical characteristics.

Parameter	All (n = 86)	Non-ON (n = 71)	ON (n = 15)	*P*
Age (year)	35.22 ± 12.42	35.96 ± 12.89	37.47 ± 10.20	0.672
Gender (male/female)	65/86	52/19	13/2	0.341
Follow up duration (years)	9.42 ± 5.59	10.80 ± 5.07	2.91 ± 2.43	<0.001
Alcoholism	18 (20.9%)	14 (19.7%)	4 (26.7%)	0.548
Major depression disorder	23 (26.7%)	4 (26.7%)	19 (26.8%)	0.994
Cumulative steroid dose (g)^+^	6.47 ± 5.27	6.44 ± 5.71	6.61 ± 2.31	0.906
Duration of steroid treatment (IQR)	28 (13–48)	27 (13–44)	39 (23–71)	0.256
Time to hospitalization (days)	19.06 ± 26.91	18.98 ± 27.14	19.43 ± 26.72	0.953
Estimated ingestion amount (mL)	56.27 ± 65.70	57.80 ± 70.13	49.00 ± 39.78	0.640
Blood paraquat level _first day_ (PPM)	1.45 ± 2.03	1.53 ± 2.11	1.07 ± 1.60	0.438
Urine paraquat level _first day_ (PPM)	28.69 ± 21.16	27.16 ± 21.21	35.85 ± 20.08	0.150
Creatinine (mg/dL) _first day_	1.39 ± 0.87	1.29 ± 0.87	1.81 ± 0.81	0.167
AST _first day_ level (U/L)	34.86 ± 14.04	36.40 ± 16.65	31.00 ± 5.65	0.687
ALT _first day_ level	34.88 ± 37.26	30.16 ± 40.31	36.60 ± 17.84	0.884
Bilirubin _first day level_ (U/L)	1.07 ± 0.62	1.44 ± 0.70	0.95 ± 0.77	0.252
PaO_2 first day_ (mmHg)	86.35 ± 12.19	86.84 ± 12.13	84.04 ± 12.65	0.422
PaCO_2 first day_ (mmHg)	34.43 ± 5.48	34.11 ± 5.43	35.91 ± 5.69	0.250
AaDO_2 first day_ (mmHg)	20.40 ± 12.05	20.32 ± 12.66	20.80 ± 8.97	0.889
AaDO_2 48-h_ (mmHg)	29.78 ± 20.12	30.16 ± 19.31	27.99 ± 24.29	0.707
HCO_3_^−^_first day_ (meq/dL)	22.34 ± 3.53	22.01 ± 3.37	23.88 ± 3.99	0.062
PaCO_2 48-h_ (mmHg)	38.69 ± 12.67	37.50 ± 7.99	44.28 ± 24.80	0.059
PaO_2 48-h_ (mmHg)	72.08 ± 18.47	73.30 ± 18.84	66.33 ± 15.92	0.186
HCO_3_^−^_48-h_ (meq/dL)	24.81 ± 3.50	24.64 ± 3.65	25.66 ± 2.64	0.307
PaO_2_/FiO_2 first day_	431.77 ± 60.96	434.22 ± 60.64	420.21 ± 63.23	0.422
PaO_2_/FiO_2 48-h_	343.26 ± 84.96	349.05 ± 89.73	315.87 ± 75.82	0.186
SIPP score	2.95 ± 3.89	2.76 ± 3.20	4.72 ± 6.94	0.404
AKIN _48-h_ score	0.47 ± 0.84	0.38 ± 0.74	0.87 ± 1.13	0.040
SOFA _48-h_ score	1.94 ± 1.68	1.76 ± 1.52	2.80 ± 2.14	0.028

AaDO_2_: alveolar-arterial differences in oxygen tension; AKIN score: The Acute Kidney Injury Network score; AST: aspartate transaminase; ALT: alanine transaminase; First day: at admission; FiO_2_: percentage of inspired oxygen; IQR: interquartile range; ON: osteonecrosis; PaO_2_: partial pressure of oxygen in arterial blood; PaCO_2_: partial pressure of carbon dioxide in the blood; PPM: parts per million; SIPP: severity index of paraquat poisoning; SOFA score: sequential organ failure assessment score; 48-h: 48 hours after admission.

^+^Cumulative steroid dose is calculated in prednisone equivalent dose for all intravenous and oral glucocorticoid.

**Table 2:** Patients data and staging of the hip.

No	Age	Comorbidity	Pathology	Side	ACRO	Interval*	Intervention
1	23	Nil	Nil	L	4	29.5	THA
2	52	Nil	Femoral head ON	R	4	25.4	THA
3	30	Hyperuricemia	Femoral head ON	B	4/4	27.7	THA
4	26	Nil	Nil	B	4/4	19.8	THA
5	28	Alcoholism	Femoral head ON	B	4/3	27.6	THA
6	31	MDD	Femoral head ON	R	4	34.2	THA
7	39	Nil	Femoral head ON	R	4	22.5	THA
8	56	Hypertension	Nil	L	4	7.7	THA
9	45	MDD Alcoholism	Femoral head ON	B	4/4	125.9	THA
10	32	MDD	Nil	L	4	12.6	THA
11	29	Amphetamine abuser Alcoholism	Nil	L	4	34.4	THA
12	34	Hypertension	Femoral head ON	B	4/3	12.2	THA
13	45	MDD	Femoral head ON	L	4	71.0	THA
14	46	Hypertension	Femoral head ON	R	4	30.4	THA
15	46	Alcoholism	Femoral head ON	R	4	44.7	THA

^*^Interval from paraquat ingestion to diagnosis of osteonecrosis.

ARCO: Association of Research Circulation Osseous staging system; B: both hips; L: left hip; MDD: major depressive disorder; ON: osteonecrosis; R: right hip; THA: Total Hip Arthroplasty.

### Predictors of ON

**Figure 1:**
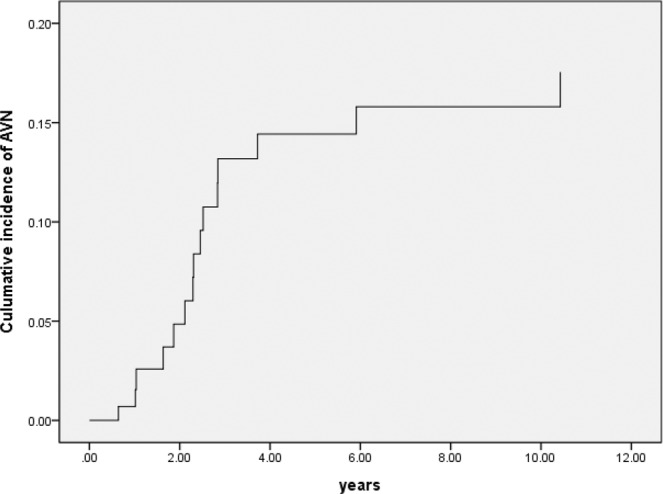
Cumulative incidence curve of femoral head osteonecrosis.

Univariate Cox regression identified several clinical variables that were significantly associated with ON (Table 3). Multivariate logistic regression analyses indicated that higher PaCO_2 48-h_ (p = 0.002), and higher AKIN _48-h_ score (p = 0.006) were independent predictors of ON. Notably, the SOFA 48-h score and serum HCO_3_ on first day were no longer significant predictors using multivariate analysis. The cumulative incidence curve showed most of ON episodes occurred within 2 to 4 years after paraquat intoxication and then plateau after 6 years (Fig. 1).

**Table 3:** Cox regression analysis for osteonecrosis of femoral head.

Parameter	β Coefficient	SE	Odds ratio (95% CI)	*P*
*Univariate*				
PaCO_2 48-h_ (mmHg)	0.036	0.014	1.037 (1.010–1.065)	0.007
HCO_3 first day_ (meq/dL)	0.127	0.064	1.135 (1.001–1.287)	0.049
AKIN _48-h_	0.528	0.231	1.696 (1.078–2.668)	0.022
SOFA _48-h_	0.301	0.126	1.351 (1.055–1.731)	0.017
*Multivariate*				
PaCO_2 48-h_ (mmHg)	0.044	0.014	1.045 (1.017–1.073)	0.002
AKIN_48-h_	0.633	0.232	1.883 (1.194–2.970)	0.006

AKIN: acute kidney injury network, SOFA: sequential organ failure assessment, SE: standard error, CI: confidence interval, AaDO_2_: alveolar–arterial differences in oxygen tension, PaO_2_ 48-h: partial pressure of oxygen in arterial blood 48 h after admission, PaCO_2_ 48-h: partial pressure of carbon dioxide in the blood 48 h after admission, eGFR first day: estimated glomerular filtration rate at admission

## Discussion

To our knowledge, this is the first retrospective study for ON of femoral head after paraquat intoxication. Previous literatures mainly focused on acute poisoning and pulmonary function of paraquat poisoning. Our study result raised the importance of this easily overlooked issue after paraquat poisoning. Present cohort data are important because large number of paraquat survivors, composed of 86 survivors out of initial 187 patients with standard detoxification protocol treating all paraquat-intoxicated patients: charcoal hemoperfusion, methylprednisolone and cyclophosphamide pulse therapies. After followed up more than 10 years, overall ON rate was 17.4%. Besides, the diagnosis of ON in our cohort was based on orthopedic referral after symptoms instead of routine screening. Most of our ON group patients are in advanced ARCO stage with crescent sign and joint destruction. The diagnosis of ON was late in our cohort. Despite the late of diagnosis and possible underdiagnosis, 14.0% of patient experienced advanced ON requiring surgery after 3 years of paraquat intoxication (Fig. 1). For comparison, in a study of systemic lupus erythematosus (SLE) required steroid treatment, only 8.9% of patients experienced symptomatic ON after 3 years of treatment^21^. In addition, most ON episodes occurred after 2 to 4 years of paraquat intoxication with mean interval 2.91 years. The plateau of ON episodes after 6 years also highly suggests ON of femoral head is related to paraquat intoxication. It is crucial information that clinician have to pay more attention for hip condition during follow-up.

More than 20 percent of our patients suffered from major depressive disorder, and chronic pain is prevalent among them, making the diagnosis of ON difficult^22^. As mentioned earlier, ON is a debilitating disease, causing marked burden not only physically but also psychologically. In our previous study, mood disorders were common among self-poisoning paraquat patients^1^. There is also reported increasing psychological distress in ON patients^7^. Surgical intervention substantially contributed to relieving pain and improving hip function in patients with ON of the hip joint^23^. Vigilant monitoring and early diagnosis of ON of femoral head are more important than general population in order to prevent further physiological and psychological distress^1,7^.

The patients in the ON group were more severe intoxicated than non-ON patients. Higher SOFA _48-h_ scores and AKIN _48-h_ scores were noted ON patients than non-ON patients. While using multivariate regression, clinically useful parameters such as PaCO_2 48-h_ and AKIN _48-h_ score were powerful predictors of ON. In our previous study, SOFA _48-h_ scores help to predict mortality after severity index of paraquat poisoning (SIPP) score with AUROC 0.7956 ± 0.033. SIPP scores is powerful predictor for AKI, and non-survivors of paraquat intoxication usually have higher PaCO_2_ at 48 hours after admission^24,25^. While treatment is standardized, it is reasonable to assume that ON survivors have more severe paraquat intoxication than their non-ON counterparts. There are several possible mechanisms. First, paraquat intoxication generates reactive oxygen species such as superoxide anions, hydrogen peroxide and hydroxyl radical, leading to cell death^11,26^. Primary cell death of subchondral bone is also a proposed pathogenesis for ON^9^. Second, paraquat promoted receptor activator for nuclear factor κ B ligand (RANKL) expression, causing subsequent impairment of canalicular network and bone lost^10^. Moreover, mitochondrial superoxide overproduction after paraquat treatment impaired chondrocyte extracellular matrix homeostasis^11^. However, there is no statistical difference in SIPP score and blood paraquat level between ON and non-ON groups in this study. Notably, patient with severe paraquat intoxication with very high SIPP score were unlikely to survive acute intoxication phase, and such patient would not be able survive long enough to experience long term complication such as ON and these patients were not included in our study, causing statistically insignificance. Interestingly, in the same cohort, AUROC of SOFA _48-h_ scores for predicting mortality after paraquat intoxication is higher than AKIN _48-h_ (0.795 and 0.671, respectively)^25^. In addition, urine paraquat level is slightly higher in the ON group than non-ON group (35.85 ± 20.08 vs 27.16 ± 21.21, p = 0.150).

Glucocorticoid may also play a role in developing ON in this cohort as well. Indeed, for preventing lung fibrosis and better chance of survival, pulse steroid therapy was given routinely in our facility along with early hemoperfusion, and cyclophosphamide. According to detoxification protocol, we tapered steroid as soon as the patient’s clinical condition stabilized. Paraquat intoxication is usually single episode, and glucocorticoid treatment in our paraquat detoxification protocol is relatively short, with median duration of treatment only 28 days, unlike in rheumatic disease which may need long term steroid treatment. Clinician’s decision to tapper glucocorticoid is based on extent of lung fibrosis, hypoxia, or dyspnea of survivors. Same peak glucocorticoid dose of 1000 mg/day was used in both ON and non-ON group. Both groups also have similar cumulative glucocorticoid dose. Slightly longer exposure duration was noted in the ON group than non-ON group. However, the difference didn’t reach statistical significance. The exact pathogenesis of glucocorticoid associated ON is still under debate. Arterial microemboli caused by alteration in lipids, blocked venous blood flow by increased adipocyte size and number in the bone marrow compartment, and increased intraosseous pressure due to venous endothelial cell change had all been proposed for possible mechanism^27–29^. One study reported that pulse steroid increases the risk of ON in systemic lupus erythematous (SLE) patients, whereas others have failed to report such association^30–33^. However, studies had shown positive correlation between mean daily glucocorticoid dose and ON in post renal transplant patients and SLE patients^30,34–37^. Although similar exposure in both groups, glucocorticoid may participate in pathogenesis of ON in this cohort. Combining possible concern about ON of paraquat and steroid, clinician should tapper steroid as soon as possible. Further study is still needed for evaluate the risk and benefit of glucocorticoid after paraquat intoxication.

Recent RCT evaluating effectiveness of high-dose immunosuppression therapy for paraquat intoxication had great impact on toxicology. Researchers reported high-dose immunosuppression dose not improve survival in paraquat-poisoned patients. However, none of the patients in such trial received hemodialysis or hemoperfusion. Previous study reported hemoperfusion appears to be an indispensable treatment for patients with acute paraquat poisoning, which may cause the poor survival rate in patients who survived more than 6 days in the immunosuppression arm^38^. Timeframe in which the increased elimination will have an impact on the distribution into tissue is very short^39^. Though planned review for hemoperfusion has not yet been completed by Extracorporeal Treatment In Poisoning (EXTRIP), there may never be a well-designed evidence based study in the management of paraquat poisoning because of its urgent need for treatment and somewhat obscure nature^40,41^. Due to severity of paraquat poisoning exposure and lack of life-saving alternatives, extracorporeal removal would be important for paraquat intoxication^5,42^. Early hemoperfusion may improve survival, if the patient received hemoperfusion in less than 5 hours^5^. Nationwide study in Taiwan also reported better survival in immunosuppression with hemoperfusion for paraquat-poisoned patients than hemoperfusion alone. The best survival effect of immunosuppression is the combination of methylprednisolone, cyclophosphamide and daily dexamethasone, especially in patients with younger age^43^. Paraquat is known to selectively accumulated in the lung and the systemic toxicity is dominated by lung toxicity. It initially induced destructive phase of lung followed by proliferative phase. Destructive phase usually occurs within 1–3 days of intoxication. Inflammatory response arises during destructive phase and will maintain throughout the proliferative phase. Followed by destructive phase is proliferative phase, when extensive fibrosis and severe anoxia take place in order to repair extensive lung damage^44^. Intra-alveolar fibrosis with subsequent obliteration of alveoli is more important than interstitial fibrosis for paraquat poisoning. Intra-alveolar migration of interstitial cells, which will differentiate into myofibroblasts and smooth-muscle cell plays an important role in this process^45^. Based on this two-phases pathophysiology, combination both removal of culprit by hemoperfusion and immunosuppression is reasonable treatment strategy to reduce paraquat pulmonary toxicity. Without removal of culprit, immunosuppression therapy alone would possibly not have benefit, considering the high degree of damage caused by paraquat. Indeed, leukocyte suppression by corticosteroid and cyclophosphamide had been proposed to treat paraquat poisoning as early as 1986^46^. Several studies had showed the better survival treated with hemoperfusion plus immunosuppression than hemoperfusion alone^4,6,43,47,48^. In rat study, cyclophosphamide is effective for reducing the severity of paraquat-induced lung injury, possibly by modulating superoxide dismutase, catalase, and TGF-β1 levels^49^. With high mortality rate of paraquat intoxication, we still recommend hemoperfusion and immunosuppression along with standard detoxification protocol. Though ON related to cyclophosphamide had been reported in some literature, most are used in conjunction with steroid or in children, which were excluded in our study^50,51^.

There are several limitations in our study. First, there is only 2 case reports in the previous literature regarding ON of femoral head after paraquat intoxication, and hip examination was not routinely done. The diagnosis of ON was based on orthopedic referral after symptoms instead of routine screening. The diagnosis of ON was late in our cohort, and underestimation of early stage of ON was likely. However, to predict the exact possibility paraquat induced ON is not the focus of this article, but first to raise alarm of this overlooked long-term complication after paraquat intoxication. Meanwhile, orthopedic follow up in asymptomatic paraquat patients is reasonable for early diagnosis of ON. Second, it is not clear the role of glucocorticoid and alcoholism in our study. Both ON and non-ON group were treated with similar amount and duration of glucocorticoid and had similar percentage of underlying alcoholism. It is not clear that paraquat act as a direct culprit for ON or an aggravation factor for glucocorticoid or alcoholism induced ON. Further investigation of exact pathophysiology is needed. Last, this is only a single-centered study in Taiwan. However, our cohort was derived from a large tertiary hospital with 3700 beds with 12 years follow up, and the result from our cohort is the starting point of reasrch regarding paraquat and ON.

## Conclusion

In summary, ON of femoral head is an easily overlooked complication after paraquat intoxication, involving 17.4% of survivors. Most of ON episodes occurred within 2 to 4 years after paraquat intoxication and then plateau after 6 years. ON patients exhibited higher SOFA and AKIN scores than non-ON patients. Higher AKIN_48-h_ score and higher PaCO_2 48-h_ after admission significantly predicted ON of femoral head after paraquat intoxication. However, due to ethical issue, randomized control trial is not feasible for toxicology study. Besides, this study was single-center retrospective, included a small population of patients and involved a short period of follow-up, further studies are warranted to confirm our results.

## Materials and Methods

### Ethics statement

This retrospective observational study was designed according to the guidelines of the Declaration of Helsinki. Because this study involved the retrospective review of delinked existing data, specific informed consent was exempted by the Medical Ethics Committee of Chang Gung Memorial Hospital and the Institutional Review Board (IRB). The trial was approved by IRB with approval number 201900758B0. Same published cohort study had been retrospectively analyzed for evaluating acute paraquat toxicity^5,14,25^. All data were securely protected by the elimination of identifying information from the main data sets, disclosed only to the investigators and analyzed anonymously. All primary data were collected by procedures outlined in epidemiology guidelines in order to strengthen the reporting of observational studies.

### Patients

The study was held in Chang Gung Memorial Hospital, Linkou branch, a 3700-bed tertiary referral medical center situated in northern Taiwan. Total 187 patients were referred because of intentional paraquat ingestion between January 2000 and December 2012, and 86 of them survived after acute paraquat intoxication.

### Inclusion and exclusion criteria

Patients were included in this study if they were >18 years old with paraquat intoxication history. Urine paraquat test was performed in these patients to screen paraquat level and was included if more than 5 ppm. Dermal and intravascular paraquat exposure were excluded in this study^47,52^. We also excluded the patients with nondetectable paraquat level in both urine and blood, with other major comorbidities, such as cancer, heart, lung, diseases, or serum concentration of ALT > 36 mg/dL, total bilirubin >3 mg/dL, or creatinine >1.2 mg/dL. Diagnoses of major comorbidities were based on comprehensive clinical, physical, and laboratory examinations.

### Diagnosis of paraquat poisoning

Prompt treatment is crucial for paraquat intoxication and presumptive diagnosis of paraquat poisoning was based majority on history of poison and urine sodium dithionite screen test. Such test is the reduction of paraquat by sodium dithionite under alkaline conditions to form stable, blue-colored radical ions^53^. The urine test was used as a paraquat screen and the results was available within 30 minutes in Chang Gung Memorial Hospital, Linkou branch. The confirmatory diagnosis of paraquat poisoning was the analysis of the blood paraquat concentration (spectrophotometry, Hitachi, Tokyo, Japan), which needs at least 4 hours waiting for the results in our facility.

### Protocol for paraquat detoxification

Protocol for paraquat detoxification had been well established in our institution^4,5^. Gastric lavage via nasogastric tube with a large amount of 0.9% was given to the intoxicated patient, followed by 1 g/kg activated charcoal with 250 mL magnesium citrate. We routinely perform charcoal hemoperfusion with a charcoal-containing (Adsorba, Gambro, Germany) dialysis machine (Surdial, Nipro, Japan) as long as the urine paraquat concentration more than 5 ppm^5^. Additional session of hemoperfusion was arranged if the urine paraquat concentration was still more than 5 ppm 4 hours after the first hemoperfusion. High intensity immunosuppression was also given after hemoperfusion with methylprednisolone (1 g/day) for three days and pulse therapies of cyclophosphamide (15 mg/kg/day) for two days^4^. Intravenous dexamethasone (20 mg/day) was administered for another 11 days after methylprednisolone pulse therapy then tapered according to patient’s clinical condition. Cyclophosphamide and methylprednisolone were administered after the extracorporeal treatment for preventing potential removal. If the patient experienced severe hypoxemia (i.e. PaO_2_ was <60 mmHg), repeated pulse therapies with cyclophosphamide and methylprednisolone were given to the patient with the duration more than two weeks after the initial treatment, unless the patient had leucopenia (white cell count <3000/m^3^). After pulse therapy, steroid was then soon tapered to oral form according to patient’s clinical condition. Cumulative steroid dose was calculated as prednisone equivalent dose for all intravenous and oral steroid. We avoid extra oxygen supply throughout their hospitalization. In order to prevent free radical related acute lung injury and systemic toxicity^54^.

### Diagnosis of ON of femoral head

While the patients experienced hip pain, they will be refereed to orthopedic surgeon for evaluation. MRI is currently the most sensitive tool for diagnosing ON, and orthopedic surgeons routinely used MRI for preoperative surgical evaluation^55–57^. Surgical type was chosen based on clinical context surgeon’s expertise. Since our study patients of paraquat intoxication were relatively young and had less comorbidity, all patients with ON received surgical treatment.

### Definition of sequential organ failure assessment (SOFA), and AKIN scores

Data were collected and assessed as baseline demographics, while SOFA and AKIN scores 48 hours after admission (SOFA _48-h_ and AKIN _48-h_). Nadir PaO_2_ for each patient was also recorded. The SOFA score is composed of six variables: PaO_2_/ FiO_2,_ platelet, total bilirubin, mean arterial pressure, Glasgow coma scale, creatinine. These variables representing respiratory, coagulation, liver, cardiovascular, neurological and renal systems. Each organ system is scored from 0 (normal) to 4 (high degree of dysfunction/failure)^25^. The AKIN criteria classify AKI into three stages of severity (stages 1, 2, and 3)^14^. Stage 1 is defined as any of increasing creatinine ≥0.3 mg/dL or elevation ≥150 to 200% of baseline or decreasing urine output to 0.5 mL/kg/h for more than 6 hours. Stage 2 is defined as any of increasing creatinine >200 to 300% or decreasing urine output to 0.5 mL/kg/h for more than 12 hours. Stage 3 is defined as any of increasing creatinine >300%, baseline creatinine ≥4 mg/dL, decreasing urine output to 0.3 mL/kg/h for more than 24 hours or anuria for 12 hours.

### Definition of severity index of paraquat poisoning (SIPP) score

The SIPP score is calculated as serum paraquat concentration (ppm) × the time to treatment (hours)^58^.

### Statistical analysis

All data were analyzed by SPSS 20.0 for windows (SPSS, Inc., Chicago, IL, USA). All continuous parameters were assessed by the Kolmogorov-Smirnov test for normal distribution. Descriptive statistics including mean, standard deviation, and percentage were calculated for continuous variables. Student’s t test was used for comparing the means of continuous variables and normally distributed data, while Mann-Whitney U test was used for non-normally distributed data. Chi-square test was used for analyzing categorical parameters. Univariate logistic regression analysis was used for assessing risk factors and multiple Cox regression with forward elimination was applied in multivariate analysis. All statistical tests were two tailed and statistically significant was defined as p < 0.05.
